# Predictors of low birth weight and preterm birth in rural Uganda: Findings from a birth cohort study

**DOI:** 10.1371/journal.pone.0235626

**Published:** 2020-07-13

**Authors:** Jorick Bater, Jacqueline M. Lauer, Shibani Ghosh, Patrick Webb, Edgar Agaba, Bernard Bashaasha, Florence M. Turyashemererwa, Robin Shrestha, Christopher P. Duggan

**Affiliations:** 1 Department of Global Health and Population, Harvard T.H. Chan School of Public Health, Boston, Massachusetts, United States of America; 2 Division of Gastroenterology, Hepatology and Nutrition, Boston Children's Hospital, Boston, Massachusetts, United States of America; 3 USAID Feed the Future Innovation Lab for Nutrition at Tufts University, Boston, Massachusetts, United States of America; 4 Gerald J. and Dorothy R. Friedman School of Nutrition Science and Policy at Tufts University, Boston, Massachusetts, United States of America; 5 Department of Agribusiness and Natural Resource Economics, Makerere University, Kampala, Uganda; 6 School of Food Technology and Nutrition, Makerere University, Kampala, Uganda; 7 Department of Nutrition, Harvard T.H. Chan School of Public Health, Boston, Massachusetts, United States of America; African Population and Health Research Center, KENYA

## Abstract

**Background:**

Approximately 20.5 million infants were born weighing <2500 g (defined as low birthweight or LBW) in 2015, primarily in low- and middle-income countries (LMICs). Infants born LBW, including those born preterm (<37 weeks gestation), are at increased risk for numerous consequences, including neonatal mortality and morbidity as well as suboptimal health and nutritional status later in life. The objective of this study was to identify predictors of LBW and preterm birth among infants in rural Uganda.

**Methods:**

Data were derived from a prospective birth cohort study conducted from 2014–2016 in 12 districts across northern and southwestern Uganda. Birth weights were measured in triplicate to the nearest 0.1 kg by trained enumerators within 72 hours of delivery. Gestational age was calculated from the first day of last menstrual period (LMP). Associations between household, maternal, and infant characteristics and birth outcomes (LBW and preterm birth) were assessed using bivariate and multivariable logistic regression with stepwise, backward selection analyses.

**Results:**

Among infants in the study, 4.3% were born LBW (143/3,337), and 19.4% were born preterm (744/3,841). In multivariable analysis, mothers who were taller (>150 cm) (adjusted Odds Ratio (aOR) = 0.42 (95% CI = 0.24, 0.72)), multigravida (aOR = 0.62 (95% CI = 0.39, 0.97)), or with adequate birth spacing (>24 months) (aOR = 0.60 (95% CI = 0.39, 0.92)) had lower odds of delivering a LBW infant Mothers with severe household food insecurity (aOR = 1.84 (95% CI = 1.22, 2.79)) or who tested positive for malaria during pregnancy (aOR = 2.06 (95% CI = 1.10, 3.85)) had higher odds of delivering a LBW infant. In addition, in multivariable analysis, mothers who resided in the Southwest (aOR = 0.64 (95% CI = 0.54, 0.76)), were ≥20 years old (aOR = 0.76 (95% CI = 0.61, 0.94)), with adequate birth spacing (aOR = 0.76 (95% CI = 0.63, 0.93)), or attended ≥4 antenatal care (ANC) visits (aOR = 0.56 (95% CI = 0.47, 0.67)) had lower odds of delivering a preterm infant; mothers who were neither married nor cohabitating (aOR = 1.42 (95% CI = 1.00, 2.00)) or delivered at home (aOR = 1.25 (95% CI = 1.04, 1.51)) had higher odds.

**Conclusions:**

In rural Uganda, severe household food insecurity, adolescent pregnancy, inadequate birth spacing, malaria infection, suboptimal ANC attendance, and home delivery represent modifiable risk factors associated with higher rates of LBW and/or preterm birth. Future studies on interventions to address these risk factors may be warranted.

## Background

Low birthweight (LBW) (defined as <2500 g) among newborn infants (which includes those born preterm (<37 weeks gestation), with intrauterine growth restriction (IUGR), or both), is a significant predictor of neonatal mortality and morbidity as well as future health and nutritional status [1, 2]. Despite being a public health priority for decades, progress in reducing the number and prevalence of infants born LBW has been limited. Today, an estimated 20.5 million (14.6%) infants globally are born LBW, primarily (>90%) in low- and middle-income countries (LMICs), mainly in southern Asia and sub-Saharan Africa [3]. In sub-Saharan Africa, specifically, the number of LBW live births increased from 4.4 million in 2000 to 5.0 million in 2015 [3]. In Uganda, 12% of infants are born LBW [4], 14% of infants are born premature [5], and neonatal mortality has stagnated over the past decade (27 deaths/1,000 live births) [6].

Across the globe, including in Uganda, persistent high rates of LBW continue to hinder global efforts to reduce infant mortality and improve child growth outcomes. Overall, it is estimated that LBW is a significant underlying factor in >80% of neonatal deaths (i.e, death <28 days after birth) [7]. In Uganda, preterm birth is the main cause of an estimated 28% of neonatal deaths [8]. According to a 2012 meta-analysis incorporating studies from Uganda, Kenya, and Tanzania, the odds of neonatal death are seven times higher for LBW infants compared to non-LBW infants [9]. In addition, LBW infants who survive infancy experience a 2.5 to 3.5-fold higher odds of wasting, stunting, and underweight [1] as well as delayed and/or diminished neurodevelopment [10, 11]. Finally, intrauterine programming and genetic modulation associated with LBW are postulated to increase risk of chronic diseases, including obesity, hypertension, and insulin resistance later in life [12, 13].

Given the serious health implications of LBW, United Nations Member States endorsed the target of a 30% reduction in LBW globally between 2012 and 2025 during the 65th World Health Assembly (WHA), [14]. However, progress towards this goal has been impeded by a lack of understanding as to the underlying predictors of adverse birth outcomes, which broadly include factors related to genetics, maternal health and nutritional status, environmental exposures, and access to antenatal care (ANC) during pregnancy. Furthermore, an overall paucity of data on the prevalence of LBW and preterm birth in LMICs like Uganda as well as observed regional and sub-regional variation in both rates and underlying predictors make further studies warranted. This analysis uses data collected from a prospective birth cohort study to identify the household, maternal, and infant risk factors associated with LBW and preterm birth in rural northern and southwestern Uganda.

## Methods

### Approvals

Study approval was obtained from the Makerere University Research Ethics Committee at the School of Public Health in Kampala, Uganda; the Uganda National Council for Science and Technology in Kampala, Uganda; the Tufts Health Sciences Institutional Review Board in Boston, MA; and the Institutional Review Board at Harvard T.H. Chan School of Public Health, Boston, MA. Before enrollment into the study, written informed consent was obtained from all participants.

### Study design

The Uganda Birth Cohort Study (UBCS, NCT04233944) was a prospective birth cohort study conducted from 2014–2016 in 12 districts/16 sub-counties in rural northern and southwestern Uganda. The study, which enrolled 5,044 pregnant women, was designed to assess the impact of the Uganda Community Connector Program (UCCP), a five-year agriculture, livelihoods, and nutrition program funded by the United States Agency for International Development (USAID) which aimed to improve the nutritional status of women and children and the livelihoods of vulnerable populations in rural Uganda.

The enrollment period for the UBCS lasted approximately 12 months. Eligible women, who were identified as pregnant from a urine pregnancy test administered by village health team (VHT) workers, were referred to study staff for enrollment into the main study. Following enrollment, which occurred primarily during the second or third trimester of pregnancy, mother-infant pairs were prospectively followed every three months until infants reached six or nine months of age. Data collected in the UBCS included information on demographics and household characteristics [e.g., water, sanitation and hygiene (WASH) practices, food security, agricultural production; and gender dynamics], maternal dietary intake and diversity, pregnancy history and outcomes, breastfeeding and complementary feeding, and infant morbidity and mortality. Maternal and infant anthropometry, including infant birth weight, were also collected.

### Sample size and eligibility

Pregnant women 15–49 years of age were eligible to participate in the UBCS if they planned to reside in the study area through the completion of follow-up and provided written informed consent. The inclusion, exclusion, referral, and termination criteria for the UBCS are presented in **S1 Table**. The target enrollment for the UBCS was 5,152 pregnant women (i.e., 322 pregnant women in each of the 16 participating sub-counties). This sample size allowed for a detection of a 0.14-unit difference in child length-for-age Z-score (LAZ) (the primary outcome variable of the parent study) with 80% power and a 0.05 level of significance, assuming 30% attrition between enrollment of pregnant women and delivery for reasons such as maternal death, fetal loss, household migration, temporary relocation of the mother for delivery, withdrawal, and loss to follow-up. Furthermore, it assumed 10% attrition among live births between delivery and completion of follow-up.

**Fig 1:**
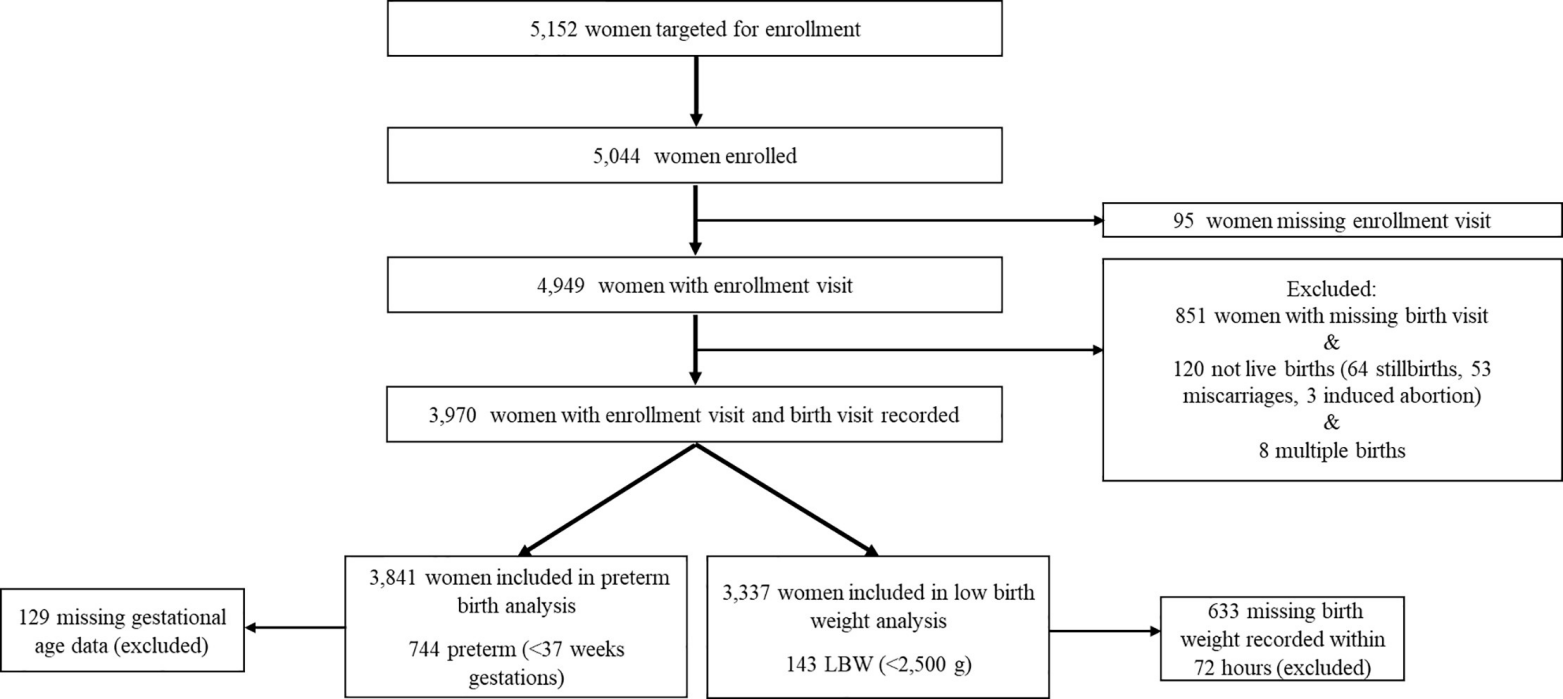
Study profile for prospective birth cohort study of pregnant women in northern and southwestern Uganda.

**Fig 1** shows the study profile for this analysis. In total, 5,044 women met the eligibility criteria and were enrolled into the UBCS. Of these, women were excluded from this analysis if they had a missing enrollment visit (n = 95) or a missing birth visit (n = 851). Furthermore, they were excluded if their infant was not born alive (n = 120) or if they had a multiple birth (n = 8). Women were excluded from the LBW analysis if birth weight of the newborn was not recorded within 72 hours (n = 633) after birth and from the preterm birth analysis if gestational age data were missing (n = 129). After exclusion criteria were applied, a total of 3,337 women were included in the LBW analysis and 3,841 in the preterm birth analysis. **S2 Table** presents the breakdown of enrollment by region, district, and sub-county for both the UBCS and this analysis.

### Data collection

The UBCS questionnaires consisted of 13 modules which were programmed onto handheld Android devices using Open Data Kit (ODK) software. Trained enumerators conducted household visits every three months from the time of enrollment until the child reached six or nine months of age. With the exception of pregnancy and birth outcome characteristics, data for this analysis came from the UBCS questionnaire administered at the enrollment visit, which occurred during the second or third trimester of pregnancy.

Household food security status was assessed using the Household Food Insecurity Access Scale (HFIAS) [15], a validated tool for use in populations across different cultural contexts, including in rural East Africa [16]. The HFIAS covers a recall period of 30 days and consists of two types of questions: nine "occurrence" and nine "frequency-of-occurrence" questions. The respondent is first asked if a given condition was experienced (yes/no) and, if it was, then with what frequency (rarely, sometimes, or often). The resulting responses can be transformed into either a continuous or categorical indicator of food security. Categorically, households are characterized into four distinct categories: food secure, mildly food insecure, moderately food insecure, or severely food insecure.

Dietary diversity during pregnancy was assessed from dietary recall data collected using the Food and Agriculture Organization (FAO) Minimum Dietary Diversity for Women (MDD-W) index [17]. Scores were computed as the sum of 10 food groups (grains, white roots and tubers, and plantains; legumes; nuts and seeds; dairy; meats, poultry and fish; eggs; vitamin A rich dark green vegetables; other vitamin A rich fruits and vegetables; other vegetables; and, other fruits) based on whether or not they were consumed in the previous 24-hours.

At the enrollment visit, tests for maternal malaria infection and hemoglobin status in pregnancy were conducted by trained nursing staff at participants’ households. Malaria infection was diagnosed using a rapid diagnostic test (RDT, SD Bioline Malaria Ag P.f/Pan test, Standard Diagnostics, Inc., Republic of Korea), and hemoglobin levels were measured using a portable hemoglobinometer (HemoCue 301; HemoCue America, Brea, CA, USA). Depending on the results, appropriate counseling, treatment, and/or referral to local health facilities were provided in accordance with UBCS standard operating procedure (SOP).

Gestational age was calculated from the first day of mothers’ last menstrual period (LMP). Maternal height was measured to the nearest 0.1 cm using a portable height board (ShorrBoard® infant/child/adult portable height-length measuring board; Weigh and Measure, LLC, Olney, MD). Infant birth weight was measured within 72 hours to the nearest and 0.1 kg using an electronic scale (Seca model 874, Seca Corporation, Hanover, MD). In all anthropometric measures, triplicate measurements were averaged to provide a single measurement.

### Statistical analysis

For the purpose of this analysis, infants born <2,500 grams were considered LBW, and infants born <37 weeks gestation were considered preterm. Adolescent pregnancy was defined as <20 years old. A binary indicator for adequate dietary diversity (i.e., ≥5 food groups in the previous 24 hours) was calculated per FAO’s recommendation [17]. Per recommendations from the World Health Organization (WHO), adequate birth spacing was defined as >24 months [18] and adequate ANC care was defined as ≥4 visits per the previous four-visit ANC (FANC) model [19].

Prior to regression analyses, categorical summary statistics for household (location, household head sex, household head marital status, household head education, household food security, water source, UCCP participation), maternal (age, height, education, dietary diversity, gravida, birth spacing, ANC visits, deworming medication, iron tablets, hemoglobin, malaria status, HIV status), and infant (sex, location of delivery) characteristics were cross tabulated among LBW and non-LBW infants and among preterm and non-preterm infants.

Bivariate logistic regression analyses were conducted to identify the relationship between independent household, maternal, and infant characteristics and the birth outcomes of interest (i.e., LBW and preterm birth). All variables in the bivariate analysis were considered for multivariable logistic regression analysis. Backward stepwise logistic regression models, which produced adjusted odds ratios (aORs), with a 0.05 cut-off for inclusion, were used to test for the predictors of LBW and preterm birth. All analyses were conducted using STATA 15 software (Stata Corps, College Station, TX, USA). In all cases, p<0.05 was considered statistically significant.

## Results

### Household, maternal, and infant characteristics

Half of the study households were located in the northern region of Uganda, and half were located in the southwestern region. Household heads were overwhelmingly male and either married or cohabitating. Mothers were on average 27 years old, 159 cm tall, and the majority had, at most, a primary school level of education (<8 years). Furthermore, a majority were multigravida, with adequate birth spacing (>24 months), and reported taking deworming medication and iron tablets during pregnancy.

**Fig 2:**
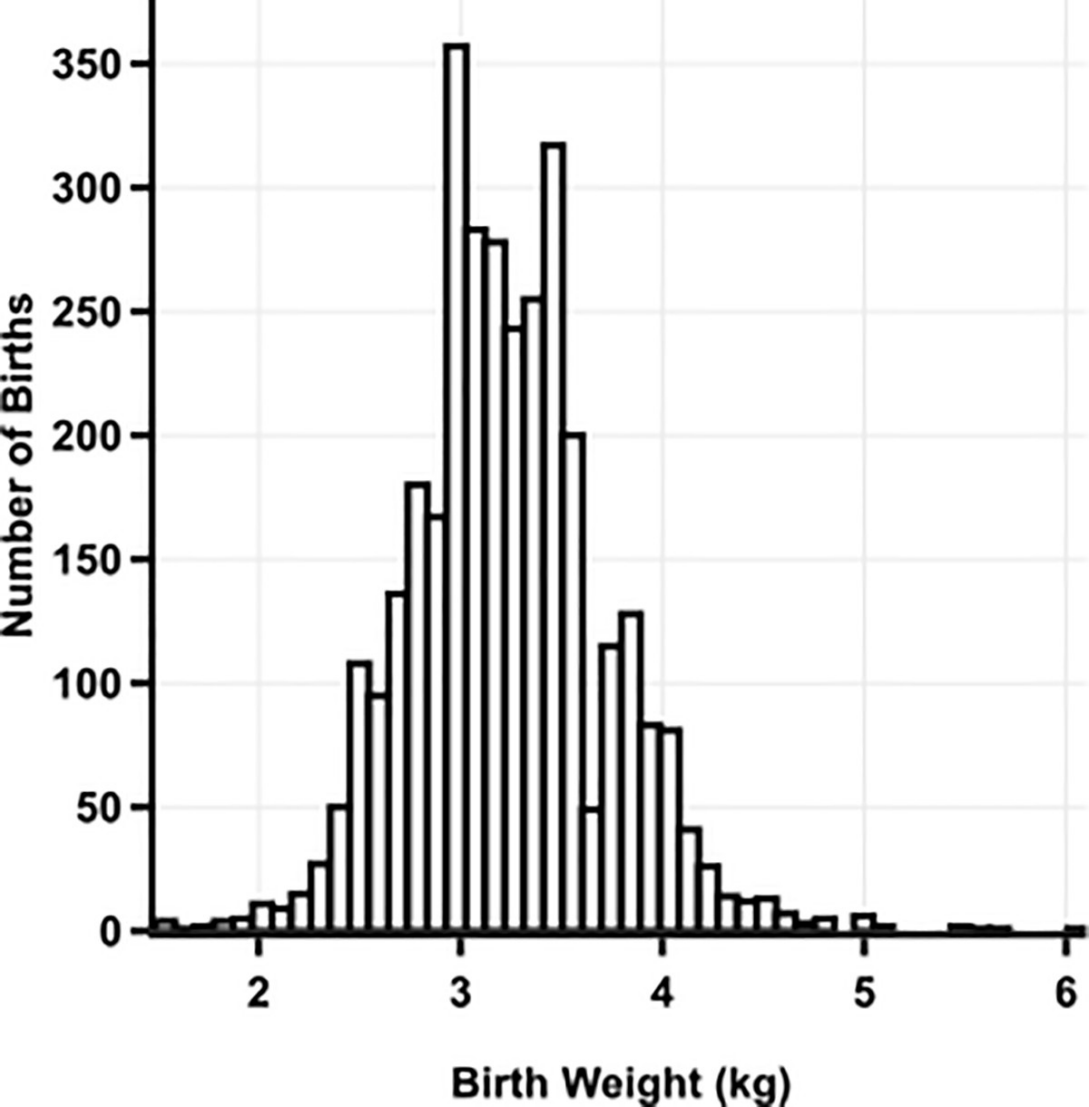
Distribution of birth weights for 3,337 infants participating in the Uganda Birth Cohort Study (UBCS)^1^.

Half of infants were male, and about one-third were delivered at home. **Fig 2** shows a distribution of birth weights. Among the 3,337 infants included in the birth weight analysis, mean birth weight was 3.2 ± 0.5 kg, and 4.3% were classified as LBW (<2500 g). Furthermore among 3,841 infants included in the analysis, 19.4% were born preterm (<37 weeks gestation).

### Predictors of LBW

In bivariate analyses (**Table 1**), being taller (>150 cm) and attending ≥4 ANC visits were associated with a significantly lower odds of delivering a LBW infant. In addition, severe household food insecurity and malaria infection during pregnancy were associated with a significantly higher odds of LBW.

**Table 1:** Household, maternal, and infant characteristics (n (%)) and their association with low birth weight (LBW) for 3,337 infants from northern and southwestern Uganda.

Characteristic	Birthweight <2500 g n = 143	Birthweight ≥2500 g n = 3,194	Crude OR (95% CI)	p-value
***Household characteristics***				
Location				
North	71 (49.7%)	1,674 (52.4%)	ref	
Southwest	72 (50.4%)	1,520 (47.6%)	1.12 (0.80, 1.56)	0.52
Sex of household head				
Male	133 (93.0%)	3,000 (93.9%)	ref	
Female	10 (7.0%)	194 (6.1%)	1.16 (0.60, 2.25)	0.65
Household head’s marital status				
Married or cohabitating	135 (94.4%)	3,030 (94.9%)	ref	
Other^1^	8 (5.6%)	164 (5.1%)	1.09 (0.53, 2.27)	0.81
Household head’s education, years				
< 8	100 (60.9%)	2,376 (74.4%)	ref	
≥ 8	43 (30.1%)	818 (25.6%)	1.25 (0.87, 1.80)	0.23
Food security (HFIAS)^2^				
Other	110 (76.9%)	2,680 (84.0%)	ref	
Severely food insecure	33 (23.1%)	511 (16.0%)	1.57 (1.05, 2.34)	0.026
Water source				
Unimproved	51 (35.7%)	1,160 (36.3%)	ref	
Improved	92 (64.3%)	2,033 (63.7%)	1.03 (0.73, 1.46)	0.87
UCCP Participation				
Yes	80 (55.9%)	1,633 (51.1%)	ref	
No	63 (44.1%)	1,561 (48.9%)	0.82 (0.59, 1.15)	0.26
***Maternal Characteristics***				
Age, years				
< 20	28 (21.2%)	482 (15.7%)	ref	
≥ 20	104 (78.8%)	2,596 (84.3%)	0.69 (0.45, 1.06)	0.09
Height, cm				
≤ 150	20 (14.0%)	187 (5.9%)	ref	
>150	123 (86.0%)	2,997 (94.1%)	0.38 (0.23, 0.63)	<0.0001
Education, years				
< 8	121 (84.6%)	2,739 (85.8%)		
≥ 8	22 (15.4%)	455 (14.3%)	1.09 (0.69, 1.74)	0.70
Diet diversity (MDD-W)^3^				
< 5	68 (47.6%)	1,665 (52.1%)	ref	
≥ 5	75 (52.5%)	1,529 (47.9%)	1.20 (0.86, 1.68)	0.28
Gravida				
Primigravida	36 (25.2%)	625 (19.6%)	ref	
Multigravida	107 (74.8%)	2,569 (80.4%)	0.72 (0.49, 1.07)	0.10
Birth spacing, months				
≤ 24	40 (28.0%)	690 (21.6%)	ref	
>24	103 (72.0%)	2,504 (78.4%)	0.71 (0.49, 1.03)	0.07
Antenatal care, visits				
< 4	84 (58.7%)	1,539 (48.2%)	ref	
≥ 4	59 (41.3%)	1,655 (51.8%)	0.65 (0.46, 0.92)	0.014
Deworming medication				
Yes	106 (74.1%)	2,433 (76.2%)	ref	
No	37 (25.9%)	760 (23.8%)	1.12 (0.76, 1.64)	0.57
Iron tablets				
Yes	129 (90.2%)	2,975 (93.2%)	ref	
No	14 (9.8%)	218 (6.8%)	1.48 (0.84, 2.61)	0.18
Hemoglobin, g/dL				
< 11	24 (19.4%)	530 (18.2%)	ref	
≥ 11	100 (80.6%)	2,381 (81.8%)	0.93 (0.59, 1.46)	0.75
Malaria test result				
Negative	112 (90.3%)	2,765 (95.0%)	ref	
Positive	12 (9.7%)	147 (5.1%)	2.02 (1.09, 3.74)	0.026
HIV test result				
Negative	116 (93.6%)	2,733 (95.0%)	ref	
Positive	8 (6.5%)	144 (5.0%)	1.32 (0.63, 2.73)	0.47
***Infant characteristics***				
Sex				
Male	84 (58.7%)	1,616 (50.6%)	ref	
Female	59 (41.3%)	1,578 (49.4%)	0.72 (0.51, 1.01)	0.06
Location of birth				
Health facility	96 (67.1%)	2,339 (73.3%)	ref	
Home	47 (32.9%)	854 (26.8%)	1.34 (0.94, 1.92)	0.11

^1^Including single, widowed, divorced, separated

^2^The HFIAS covers a recall period of 30 days and consists of two types of questions: nine "occurrence" and nine "frequency-of-occurrence" questions. The respondent is first asked if a given condition was experienced (yes/no) and, if it was, then with what frequency (rarely, sometimes, or often). Using these responses, the HFIAS categorizes households as food secure, mildly food insecure, moderately food insecure, or severely food insecure.

^3^The MDD-W is computed as the sum of food groups consumed in the previous 24 hours based on the baseline prenatal dietary recall for women.

In multivariable analysis (**Table 2**), mothers who were taller (>150 cm) (aOR = 0.42 (95% CI = 0.24, 0.72)), multigravida (aOR = 0.62 (95% CI = 0.39, 0.97)), or with adequate birth spacing (>24 months) (aOR = 0.60 (95% CI = 0.39, 0.92)) had lower odds of delivering a LBW infant. Furthermore, mothers from households with severe household food insecurity (aOR = 1.84 (95% CI = 1.22, 2.79)) or who tested positive for malaria during pregnancy (aOR = 2.06 (95% CI = 1.10, 3.85)) had higher odds.

**Table 2:** Characteristics associated with low birthweight for 3,337 infants from northern and southwestern Uganda participating in a birth cohort study^1^.

Characteristic	Adjusted OR (95% CI	p-value
Food security (HFIAS)		
Other	ref	
Severely food insecure	1.84 (1.22, 2.79)	0.004
Height, cm		
≤ 150	ref	
>150	0.42 (0.24, 0.72)	0.002
Gravida		
Primigravida	ref	
Multigravida	0.62 (0.39, 0.97)	0.038
Birth spacing, months		
≤ 24	ref	
>24	0.60 (0.39, 0.92)	0.019
Malaria test result		
Negative	ref	
Positive	2.06 (1.10, 3.85)	0.024

^1^Adjusted odds ratios (95% CIs) and *p*-values determined by developing a stepwise, backward logistic regression model with a 0.05 cut-off for inclusion.

### Predictors of preterm birth

Analyses were also performed to help identify the relationship between household, maternal, and infant characteristics and preterm birth. In bivariate analyses (**Table 3**), residing in the Southwest, being older (≥ 20 years), better educated (≥ 8 years), having more dietary diversity, with adequate birth spacing, and attending ≥4 ANC visits were associated with a significantly lower odds of delivering preterm. In addition, severe household food insecurity, not taking deworming medication or iron tablets during pregnancy, and delivering at home were associated with a significantly higher odds of delivering preterm.

**Table 3:** Household, maternal, and infant characteristics (n (%)) and their association with preterm birth for 3,841 infants from northern and southwestern Uganda.

Characteristic	< 37 weeks gestation n = 744	≥ 37 weeks gestation n = 3,097	Crude OR (95% CI)	p-value
***Household characteristics***				
Location				
North	447 (60.1%)	1,467 (47.4%)	ref	
Southwest	297 (39.9%)	1,630 (52.6%)	0.60 (0.51, 0.70)	<0.001
Sex of household head				
Male	693 (93.2%)	2,917 (94.2%)	ref	
Female	51 (6.9%)	180 (5.8%)	1.19 (0.86, 1.65)	0.28
Household head’s marital status				
Married or cohabitating	696 (93.6%)	2,941 (95.0%)	ref	
Other^1^	48 (6.5%)	156 (5.0%)	1.30 (0.93, 1.82)	0.12
Household head’s education, years				
< 8	553 (74.3%)	2,260 (73.0%)	ref	
≥ 8	191 (25.7%)	837 (27.0%)	0.93 (0.78, 1.12)	0.45
Food security (HFIAS)^2^				
Other	602 (80.9%)	2,600 (84.0%)	ref	
Severely food insecure	142 (19.1%)	494 (16.0%)	1.24 (1.01, 1.53)	0.040
Water source				
Unimproved	260 (35.0%)	1,176 (38.0%)	ref	
Improved	484 (65.1%)	1,920 (62.0%)	1.14 (0.96, 1.35)	0.12
UCCP Participation				
Yes	367 (49.3%)	1,557 (50.3%)	ref	
No	377 (50.7%)	1,540 (49.7%)	1.04 (0.88, 1.22)	0.64
***Maternal Characteristics***				
Age, years				
< 20	139 (19.3%)	459 (15.4%)	ref	
≥ 20	582 (80.7%)	2,522 (84.6%)	0.76 (0.62, 0.94)	0.011
Height, cm				
≤ 150	58 (7.8%)	210 (6.8%)	ref	
>150	686 (92.2%)	2,874 (93.2%)	0.86 (0.64, 1.17)	0.34
Education, years				
< 8	657 (88.3%)	2,597 (83.9%)	ref	
≥ 8	87 (11.7%)	500 (16.1%)	0.69 (0.54, 0.88)	0.003
Diet diversity (MDD-W)^3^				
< 5	406 (54.6%)	1,527 (49.3%)	ref	
≥ 5	338 (45.4%)	1,570 (50.7%)	0.81 (0.70, 0.95)	0.010
Gravida				
Primigravida	161 (21.6%)	642 (20.7%)	ref	
Multigravida	583 (78.4%)	2,455 (79.3%)	0.95 (0.78, 1.15)	0.58
Birth spacing, months				
≤ 24	190 (25.5%)	630 (20.3%)	ref	
>24	554 (74.%)	2,467 (79.7%)	0.74 (0.62, 0.90)	0.002
Antenatal care, visits				
< 4	102 (60.4%)	1,723 (46.9%)	ref	
≥ 4	67 (39.6%)	1,948 (53.1%)	0.51 (0.43, 0.60)	<0.001
Deworming medication				
Yes	546 (73.4%)	2,390 (72.2%)	ref	
No	198 (26.6%)	706 (22.8%)	1.23 (1.02, 1.47)	0.028
Iron tablets				
Yes	675 (90.7%)	2,902 (93.7%)	ref	
No	69 (9.3%)	194 (6.3%)	1.53 (1.15, 2.04)	0.004
Hemoglobin, g/dL				
< 11	131 (20.1%)	501 (17.6%)	ref	
≥ 11	520 (79.9%)	2,351 (82.4%)	0.85 (0.68, 1.05)	0.13
Malaria test result				
Negative	611 (93.7%)	2,719 (95.4%)	ref	
Positive	41 (6.3%)	130 (4.6%)	1.40 (0.98, 2.02)	0.07
HIV test result				
Negative	603 (94.4%)	2,651 (94.6%)	ref	
Positive	36 (5.6%)	150 (5.4%)	1.06 (0.73, 1.53)	0.78
***Infant characteristics***				
Sex				
Male	379 (50.9%)	1,564 (50.5%)	ref	
Female	365 (49.1%)	1,533 (49.5%)	0.98 (0.84, 1.15)	0.83
Location of birth				
Health facility	504 (67.7%)	2,333 (75.4%)	ref	
Home	240 (32.3%)	763 (24.6%)	1.46 (1.22, 1.73)	<0.001

^1^Including single, widowed, divorced, separated

^2^The HFIAS covers a recall period of 30 days and consists of two types of questions: nine "occurrence" and nine "frequency-of-occurrence" questions. The respondent is first asked if a given condition was experienced (yes/no) and, if it was, then with what frequency (rarely, sometimes, or often). Using these responses, the HFIAS categorizes households as food secure, mildly food insecure, moderately food insecure, or severely food insecure.

^3^The MDD-W is computed as the sum of food groups consumed in the previous 24 hours based on the baseline prenatal dietary recall for women.

In multivariable analysis (**Table 4**), mothers who resided in the Southwest (aOR = 0.64 (95% CI = 0.54, 0.76)), were ≥20 years old (aOR = 0.76 (95% CI = 0.61, 0.94)), with adequate birth spacing (aOR = 0.76 (95% CI = 0.63, 0.93)), or attended ≥4 ANC visits (aOR = 0.56 (95% CI = 0.47, 0.67)) had lower odds of delivering preterm. Furthermore, mothers who were neither married nor cohabitating (aOR = 1.42 (95% CI = 1.00, 2.00)) or delivered at home (aOR = 1.25 (95% CI = 1.04, 1.51)), had higher odds of delivering preterm.

**Table 4:** Characteristics associated with preterm birth for 3,841 infants from northern and southwestern Uganda participating in a birth cohort study^1^.

Characteristic	Adjusted OR (95% CI)	p-value
Location		
North	ref	
Southwest	0.64 (0.54, 0.76)	<0.001
Household head’s marital status		
Married or cohabiting	ref	
Other^2^	1.42 (1.00, 2.00)	0.048
Age, years		
<20	ref	
≥ 20	0.76 (0.61, 0.94)	0.011
Birth spacing, months		
≤ 24	ref	
>24	0.76 (0.63, 0.93)	0.007
Antenatal care, visits		
< 4	ref	
≥ 4	0.56 (0.47, 0.67)	<0.001
Location of birth		
Health facility	ref	
Home	1.25 (1.04, 1.51)	0.018

^1^Adjusted odds ratios (95% CIs) and *p*-values determined by developing a stepwise, backward logistic regression model with a 0.05 cut-off for inclusion.

^2^Including single, widowed, divorced, separated

### Regional sub-analyses

Regional sub-analyses showed differences in risk factors for LBW and preterm birth between the North and Southwest. In the North, severe household food insecurity (aOR = 2.18 (95% CI = 1.24, 3.85)) and malaria infection during pregnancy (aOR = 2.15 (95% CI = 1.09, 4.24)) were associated with a higher odds of delivering a LBW infant. In the Southwest, mothers who were taller (aOR = 0.34 (95% CI = 0.19, 0.63)) or with adequate birth spacing (aOR = 0.59 (95% CI = 0.36, 0.97)) had a lower odds of delivering a LBW infant.

With regard to preterm birth, mothers in the North who were older (≥20) (aOR = 0.70 (95% CI = 0.53, 0.91)), attended ≥4 ANC visits (aOR = 0.49 (95% CI = 0.39, 0.61)), or with adequate birth spacing (aOR = 0.73 (95% CI = 0.56, 0.95)) had a lower odds of delivering preterm. In the Southwest, mothers who attended ≥4 ANC visits (aOR = 0.66 (95% CI = 0.51, 0.85)) had a lower odds of delivering a preterm infant; mothers who delivered at home (aOR = 1.43 (95% CI: 1.08, 1.91) had a higher odds of delivering preterm.

## Discussion

While the predictors of adverse birth outcomes, including LBW and preterm birth, are complex, multidimensional, and geographically context-specific, their identification is key to the development of future policies and programs to improve morbidity and mortality among infants in LMICs as well as to meet the global nutrition target of a 30% reduction in LBW globally between 2012 and 2025. Using data from a large birth cohort study in rural Uganda, we identified several modifiable risk factors associated with LBW and/or preterm birth, including severe household food insecurity, adolescent pregnancy, inadequate birth spacing, malaria infection, suboptimal ANC attendance, and home delivery.

The World Food Summit in 1996 defined food security as “when all people, at all times, have physical and economic access to sufficient safe and nutritious food to meet their dietary needs and food preferences for a healthy and active life” [20]. While there is substantial evidence demonstrating that both maternal underweight and poor dietary intake during pregnancy have a significant impact on infant birth outcomes [21, 22], fewer studies have examined household food security status as a risk factor. A prospective cohort study from the United States among 294 pregnant women showed that those experiencing food-insecurity are three times more likely to give birth to a LBW infant (OR = 3.2 (95% CI = 1.4, 7.2)) [23]. Furthermore, a nationally-representative, cross-sectional study from Bangladesh, which analyzed surveys from 8,753 households with a live birth between 2006 and 2011, found the odds of LBW were significantly higher in both food-insecure poor households (OR = 1.39 (95% CI = 1.11, 1.76)) and food-insecure non-poor households (OR = 1.32 (95% CI = 1.08, 1.62)) compared to the respective food-secure groups [24].

Adolescence, a period characterized by biological immaturity, i.e., incomplete anatomical and physiological development, is a relatively well-established risk factor for adverse birth outcomes. A study by Fall et al. which pooled data from five birth cohort studies (in South Africa, Brazil, Guatemala, India and the Philippines), found significant associations between younger maternal age (≤19 years) and both LBW (aOR = 1.18 (95% CI = 1.02–1.36)) and preterm birth (aOR = 1.26 (95% CI = 1.03–1.53)) compared with mothers aged 20–24 years [25]. In a systematic review and meta-analysis of 18 studies all originating from sub-Saharan Africa, young maternal age (<17 years) was associated LBW, preterm birth, pre-eclampsia/eclampsia, and maternal and perinatal mortality [26]. While we found younger maternal age to be associated with a similarly higher risk of preterm birth, we did not observe a significant association with LBW. However, multigravida (vs. primigravida), which can serve as a proxy indicator for maternal age, was significantly associated with LBW.

According to WHO, after a live birth, the recommended interval before attempting the next pregnancy is at least 24 months in order to reduce the risk of adverse maternal, perinatal, and infant outcomes [18]. Based on the evidence reviewed for this recommendation, shorter birth-to-pregnancy intervals are associated with elevated risk of infant, neonatal and perinatal mortality, LBW, small-for-gestational age (SGA), and preterm delivery [27–31]. In our study, birth spacing <24 months was associated with almost twice the risk of LBW and 1.5 times the risk of preterm birth. This finding is supported by additional studies from sub-Saharan Africa, including Sudan [32] and Ethiopia [33], which show a similar association between inadequate birth spacing and risk of LBW and preterm birth.

It is established that malaria in pregnancy, particularly *Plasmodium falciparum* infection, and associated inflammatory processes increase energy expenditure and protein catabolism causing nutritional depletion in the mother and IUGR in the fetus [34]. Overall, malaria in pregnancy is estimated to causes approximately 900,000 LBW deliveries worldwide and over 100,000 infant deaths annually [35, 36], making it the leading preventable cause of LBW in Africa. Our findings that maternal malaria nearly doubled the risk of LBW is consistent with other studies which have demonstrated a firm association with a similar order of magnitude (doubled risk) between malaria in pregnancy and LBW [37]. Interventions that promote the prevention and treatment of malaria during pregnancy, including long-lasting insecticidal nets (LLINs), intermittent preventive treatment in pregnancy (IPTp) with sulfadoxine-pyrimethamine (SP) as part of ANC services, and prompt diagnosis and effective treatment of malaria infections, may be efficacious in improving birth and subsequent growth outcomes for infants in rural Uganda.

In addition, WHO currently recommends a minimum of 8 ANC visits to “reduce perinatal mortality and improve women’s experience of care” [38]. Universally, ANC visits provide opportunities for risk identification, health education and promotion, and the prevention and management of pregnancy-related or concurrent diseases [38]. While ~99% of women in our study received some ANC, it was insufficiently frequent (mean: 3.4 visits) by current WHO standards. In our study, women with suboptimal ANC attendance (< 4 visits) had twice the odds of delivering a preterm infant, which is supported by a number of studies across a range of geographical contexts showing an association between ANC visits and pregnancy outcomes [39–43].

Finally, according to the 2016 Demographic and Health Survey (DHS), 73% of births in Uganda occur at a health facility [6], similar to our study where 74% of births occurred at a health facility. As the majority of studies examining risk factors for adverse birth outcomes in sub-Saharan Africa are facility-based, our study is one of few to look at the association between home (vs. facility-based) delivery on risk of LBW and/or preterm birth. We found mothers who practiced home-based delivery, which could indicate lack of access to health services, to be associated with a higher risk of preterm birth.

Strengths of the study include a large sample size as well as a prospective study design, with data collection beginning during pregnancy, allowing for temporal relationships between predictors and outcomes to be examined. Birth weights were measured by trained enumerators within 72 hours of delivery, and we were able to capture births that occurred both inside and outside a health facility. Limitations of the study include imprecisions associated with assessing gestational age from date of LMP and a relatively small sample of infants born LBW. Notably, this relatively small sample may even be an overestimate of LBW as birthweights were collected over a period of 72 hours, a time when newborns typically lose weight.

## Conclusion

Despites efforts to reduce the incidence of LBW, it remains a significant public health concern for the majority of LMICs, including Uganda. As meeting the target of reducing LBW by 30% between 2012 and 2025 will require more than doubling the current rate of progress [3], it is imperative to identify the most important underlying contributors. This study identifies several modifiable risk factors for LBW and preterm birth in rural Uganda that may assist in prioritizing efforts, including reducing household food insecurity, unplanned adolescent pregnancies, and malaria infection as well as promoting adequate birth spacing, ANC attendance, and facility-based delivery.

## Supporting information

S1 Table Inclusion, exclusion, referral, and termination criteria for the Uganda Birth Cohort Study (UBCS). (DOCX)

S2 Table Uganda Birth Cohort Study (UBCS) enrollment by location (region, district, and sub-county). (DOCX)

## List of abbreviations

CI: confidence interval
FAO: Food and Agriculture Organization
HFIAS: household food insecurity access scale
HIV: human immunodeficiency virus
LBW: low birthweight
MDD-W: minimum dietary diversity for women
OR: odds ratio
UCCP: Uganda Community Connector Program
