# Supplementary material for: Predictors of low birth weight and preterm birth in rural Uganda: Findings from a birth cohort study
Source: PLoS One. 2020 Jul 13;15(7):e0235626. doi: 10.1371/journal.pone.0235626 (PMC7357758; doi:10.1371/journal.pone.0235626)
Supplement: S1 Table — (DOCX) [file pone.0235626.s001.docx]

Supplementary Table 1: Inclusion, exclusion, referral, and termination criteria for the Uganda Birth Cohort Study (UBCS)

| **Type** | **Criteria** |
| --- | --- |
| Inclusion into pregnancy surveillance | Woman aged 15-49 years |
|  | Woman intends to reside in the study area through the enrollment period |
|  | Written informed consent |
| Exclusion from pregnancy surveillance | None |
| Inclusion into UBCS study | Pregnancy is confirmed via urine pregnancy test |
|  | Mother is aged 15-49 years |
|  | Mother intends to reside in the study area through completion of follow-up |
|  | Mother intends to deliver in the study area |
|  | Mother provides written informed consent |
| Exclusion from UBCS study | None |
| Immediate referral | Mother is severely malnourished during pregnancy |
|  | Mother is severely anemic during pregnancy (hemoglobin<8.5 g/dL) |
|  | Mother has acute infectious disease during pregnancy |
|  | Mother requires hospitalization during pregnancy |
|  | Mother is diagnosed with pregnancy-induced hypertension (blood pressure higher than 140/90 mm Hg) |
|  | Child has congenital anomaly at birth |
|  | Child has very low birthweight (<1500 g) |
|  | Child has sepsis following delivery |
|  | Child has respiratory distress syndrome following delivery |
|  | Child requires hospitalization during follow-up |
|  | Child has severe acute malnutrition at 6+ months of age (mid-upper arm circumference<115 mm) |
|  | Child has severe anemia during follow-up (hemoglobin<8.5 g/dL) |
| Study termination | Completion of follow-up period |
|  | Fetal loss (spontaneous or induced abortion or stillbirth) |
|  | Child death |
|  | Relocation outside study area |
|  | Withdrawal |
