# Supplementary material for: Predictors of low birth weight and preterm birth in rural Uganda: Findings from a birth cohort study
Source: PLoS One. 2020 Jul 13;15(7):e0235626. doi: 10.1371/journal.pone.0235626 (PMC7357758; doi:10.1371/journal.pone.0235626)
Supplement: S2 Table — (DOCX) [file pone.0235626.s002.docx]

Supplementary Table 2: Uganda Birth Cohort Study (UBCS) enrollment by location (region, district, and sub-county)

| **Region** | **District** | **Sub-county** | **Total UBCS Enrollment n(%)** | **Low Birthweight Analysis**  **n(%)** | **Preterm Analysis n(%)** |
| --- | --- | --- | --- | --- | --- |
| North | Apac | Aduku | 321 (6.36%) | 224 (6.71%) | 241 (6.3%) |
| North | Apac | Apac | 319 (6.32%) | 171 (5.12%) | 190 (5.0%) |
| North | Kole | Ayer | 326 (6.46%) | 184 (5.51%) | 229 (6.0%) |
| North | Lamwo | Agoro | 322 (6.38%) | 262 (7.85%) | 285 (7.4%) |
| North | Lira | Agweng | 321 (6.36%) | 241 (7.22%) | 259 (6.7%) |
| North | Nebbi | Parombo | 322 (6.38%) | 220 (6.59%) | 230 (6.0%) |
| North | Pader | Atanga | 321 (6.36%) | 186 (5.57%) | 219 (5.7%) |
| North | Zombo | Atyak | 325 (6.44%) | 257 (7.70%) | 261 (6.8%) |
| Southwest | Kabale | Ruhija | 279 (5.53%) | 213 (6.38%) | 228 (5.9%) |
| Southwest | Kabale | Nyamweru | 288 (5.71%) | 248 (7.43%) | 254 (6.6%) |
| Southwest | Kabarole | Kibiito | 323 (6.40%) | 168 (5.03%) | 247 (6.4%) |
| Southwest | Kamwenge | Bwizi | 321 (6.36%) | 240 (7.19%) | 253 (6.6%) |
| Southwest | Kanungu | Rugyeyo | 311 (6.16%) | 181 (5.42%) | 252 (6.6%) |
| Southwest | Rukungiri | Bungangari | 323 (6.40%) | 176 (5.27%) | 212 (5.5%) |
| Southwest | Rukungiri | Buyanja | 321 (6.36%) | 171 (5.12%) | 258 (6.7%) |
| Southwest | Rukungiri | Kebisoni | 304 (6.02%) | 195 (5.84%) | 223 (5.8%) |
| Totals |  |  | 5,047 | 3,337 | 3,841 (100.0%) |
